# Supplementary material for: Shelf-Life Evaluation of “San Marzano” Dried Tomato Slices Preserved in Extra Virgin Olive Oil
Source: Foods. 2021 Jul 23;10(8):1706. doi: 10.3390/foods10081706 (PMC8393553; doi:10.3390/foods10081706)
Supplement: Supplementary file 1 [file foods-10-01706-s001.zip › foods-1291946-supplementary.pdf]

Supplementary materials

Table S1. Moisture (%) and water activity of dried tomatoes samples storage in light and dark.

**Months**	**DTL**		**DTD**			
	**^Moisture (%)**	**#a_w**	**^Moisture (%)**	**#a_w**	**^Sign.**	**#Sign.**
**0**	31.66 ± 2.01^{oA}	0.63 ± 0.06^{dB}	31.66 ± 2.01^{oA}	0.63 ± 0.06^{bB}	ns	ns
**1**	31.86 ± 2.07^{nA}	0.65 ± 0.06^{cdB}	31.87 ± 2.07^{nA}	0.66 ± 0.06^{abB}	ns	ns
**2**	32.18 ± 2.00^{mA}	0.67 ± 0.07^{bcC}	32.01 ± 2.00^{mB}	0.67 ± 0.06^{abC}	*	ns
**3**	32.82 ± 2.08^{lA}	0.67 ± 0.07^{bcC}	32.66 ± 2.31^{lB}	0.67 ± 0.06^{abC}	*	ns
**4**	34.30 ± 2.34^{iA}	0.68 ± 0.06^{bcC}	32.96 ± 2.53^{iB}	0.68 ± 0.06^{abC}	**	ns
**5**	35.59 ± 2.41^{hA}	0.68 ± 0.06^{bcC}	33.18 ± 2.46^{hB}	0.68 ± 0.06^{abC}	**	ns
**6**	35.93 ± 2.49^{gA}	0.68 ± 0.07^{bcC}	33.91 ± 2.61^{gB}	0.68 ± 0.06^{abC}	**	ns
**7**	36.66 ± 2.52^{fA}	0.68 ± 0.06^{bcC}	34.10 ± 2.63^{fB}	0.68 ± 0.06^{abC}	**	ns
**8**	37.82 ± 2.60^{eA}	0.68 ± 0.06^{bcC}	34.15 ± 2.66^{eB}	0.68 ± 0.06^{abC}	**	ns
**9**	37.86 ± 2.59^{dA}	0.69 ± 0.07^{abcC}	35.73 ± 2.61^{dB}	0.68 ± 0.06^{abC}	**	ns
**10**	37.97 ± 2.61^{cA}	0.70 ± 0.08^{abC}	36.76 ± 2.63^{cB}	0.69 ± 0.07^{abC}	**	ns
**11**	40.00 ± 2.68^{bA}	0.70 ± 0.08^{abC}	36.80 ± 2.68^{bB}	0.69 ± 0.07^{abC}	**	ns
**12**	40.04 ± 2.69^{aA}	0.71 ± 0.08^{aC}	37.63 ± 2.66^{aB}	0.69 ± 0.07^{aD}	**	*
**Sign.**	**	**	**	*		

Data are reported as mean ± standard deviation (n = 3). Differences were evaluated by one-way analysis of variance (ANOVA) test completed with a multicomparison Tukey's test. Means in the same row with different capital letters differ significantly ($p < 0.05$), means in the same column with different small letters differ significantly ($p < 0.05$). ns: not significance; * Significance at $p < 0.05$, ** Significance at $p < 0.01$.

Table S2. Chlorophylls and TCC in EVOO, EVOO_DTL and EVOO_DTD samples (mg/Kg).

**Months**	**EVOO**		**EVOO_DTL**		**EVOO_DTD**			
	^Chlorophylls	#TCC	^Chlorophylls	#TCC	^Chlorophylls	#TCC	^Sign.	#Sign.
**0**	66.17±4.23^{aA}	22.45±3.33^{aB}	66.17±5.36^{aA}	22.45±2.33^{aB}	66.17±6.03^{aA}	22.45±2.23^{iB}	ns	ns
**1**	63.51±4.56^{bB}	21.25±3.12^{bF}	56.89±4.89^{bC}	22.75±2.88^{bE}	63.74±5.33^{bA}	25.78±2.21^{aD}	**	**
**2**	47.27±3.02^{cC}	20.86±3.63^{cE}	51.43±4.02^{cA}	20.21±3.03^{hF}	48.23±5.23^{cB}	24.37±2.26^{dD}	**	**
**3**	46.68±3.56^{dA}	18.95±2.89^{dF}	42.45±4.23^{dC}	19.97±3.05^{mE}	46.59±4.26^{dB}	23.77±2.25^{gD}	**	**
**4**	42.77±3.88^{eB}	17.73±2.85^{eF}	40.8±4.02^{eC}	18.16±3.11^{nE}	44.12±4.56^{eA}	23.08±2.24^{hD}	**	**
**5**	38.49±4.02^{fB}	17.45±4.02^{fF}	37.6±3.56^{fC}	18.03±3.21^{mE}	41.12±4.02^{fA}	22.17±2.29^{lD}	**	**
**6**	36.8±2.89^{gB}	16.79±3.03^{gF}	35.4±4.03^{gC}	18.14±3.56^{lE}	37.98±4.03^{gA}	22.04±2.28^{mD}	**	**
**7**	35.79±6.33^{hA}	16.39±2.02^{hF}	32.1±3.56^{hC}	19.42±2.55^{iE}	35.61±3.96^{hB}	23.12±2.27^{hD}	**	**
**8**	35.7±2.11^{iA}	15.87±2.23^{iF}	30.5±3.95^{iC}	19.76±2.34^{gE}	35.18±3.85^{iB}	23.87±1.99^{gD}	**	**
**9**	32.01±2.56^{lB}	13.47±2.56^{lF}	29.57±3.02^{lC}	20.11±2.22^{eE}	32.87±3.74^{lA}	23.95±3.03^{fD}	**	**
**10**	31.44±3.02^{mB}	12.73±2.87^{mF}	27.34±3.11^{mC}	20.57±3.02^{dE}	32.44±3.46^{mA}	24.2±3.05^{eD}	**	**
**11**	28.71±3.12^{nB}	11.96±3.02^{nF}	24.98±2.85^{nC}	21.2±3.04^{cE}	30.12±3.25^{nA}	24.56±2.23^{cD}	**	**
**12**	28.65±2.89^{oB}	11.78±3.06^{oF}	23.1±2.66^{oC}	21.35±3.22^{cE}	29.21±3.37^{oA}	24.9±2.28^{bD}	**	**
**Sign.**	**	**	**	**	**	**		

Data are reported as mean ± standard deviation (n = 3). Differences were evaluated by one-way analysis of variance (ANOVA) test completed with a multicomparison Tukey's test. Means in the same row with different capital letters differ significantly ($p < 0.05$), means in the same column with different small letters differ significantly ($p < 0.05$). ns: not significance; ** Significance at $p < 0.01$.

Table S3. ΔE*ab values during 12 months storage of EVOO, EVOO_DTL and EVOO_DTD samples.

Months	EVOO	EVOO_DTD	EVOO_DTL	Sign.
0	0.00±0.00oA	0.00±0.00nA	0.00±0.00oA	ns
1	0,77±0.03nB	0,1±0.02nC	0,98±0.02mA	**
2	2,03±0.15mB	1,39±0.12lC	2,26±0.88iA	**
3	5,39±0.86lB	4,93±0.36dC	5,65±0.75cA	**
4	5,76±0.77iB	5,34±0.45cC	6,15±0.88aA	**
5	6,53±1.02hA	4,64±0.63eC	5,99±0.73bB	**
6	6,61±0.82gA	2,69±0.57hC	5,46±0.79dB	**
7	6,68±0.45fA	1,57±0.12iC	3,16±0.55eB	**
8	6,72±0.77eA	1,08±0.09mC	2,78±0.24gB	**
9	6,82±0.56dA	2,91±0.42gB	0,92±0.04nC	**
10	6,93±0.95cA	4,18±0.23fB	1,52±0.06lC	**
11	7,01±0.93bB	10,23±0.86bA	2,6±0.12hC	**
12	7,15±0.83aB	15,03±0.97aA	3,03±0.09fC	**
**Sign.**	**	**	**	

Data are reported as mean ± standard deviation (n = 3). Differences were evaluated by one-way analysis of variance (ANOVA) test completed with a multicomparison Tukey's test. Means in the same row with different capital letters differ significantly ($p < 0.05$), means in the same column with different small letters differ significantly ($p < 0.05$). ns: not significance; ** Significance at $p < 0.01$.

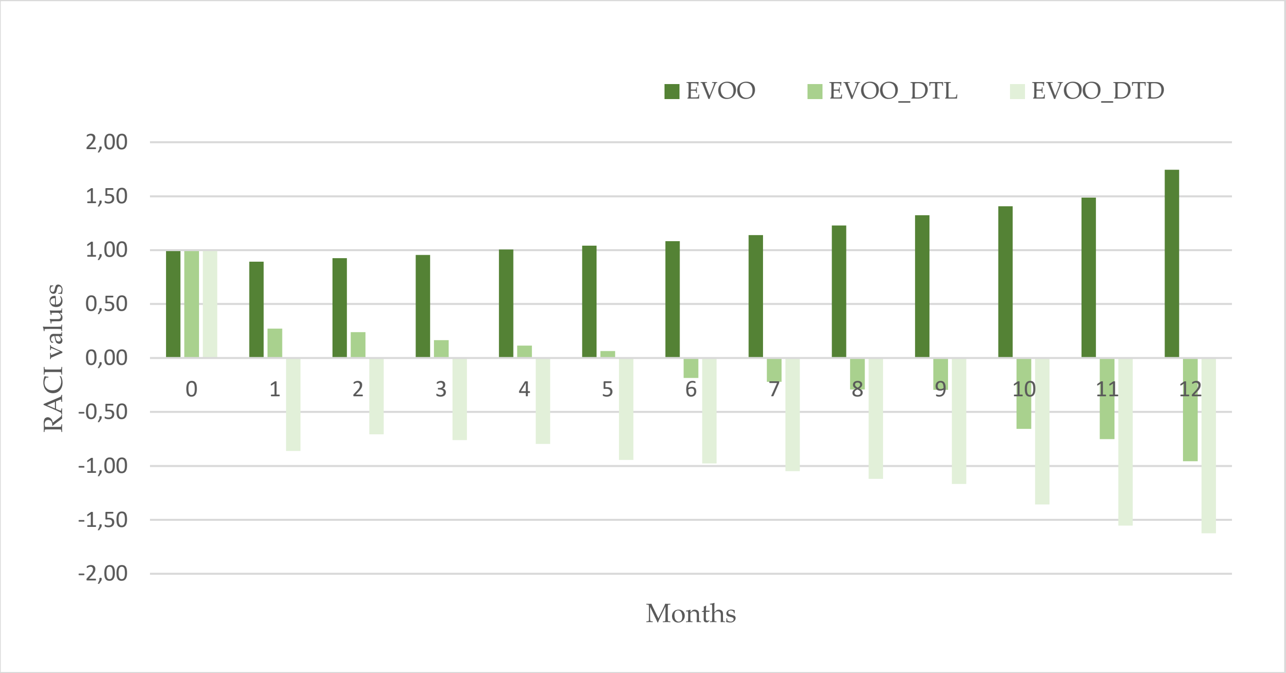

(a)

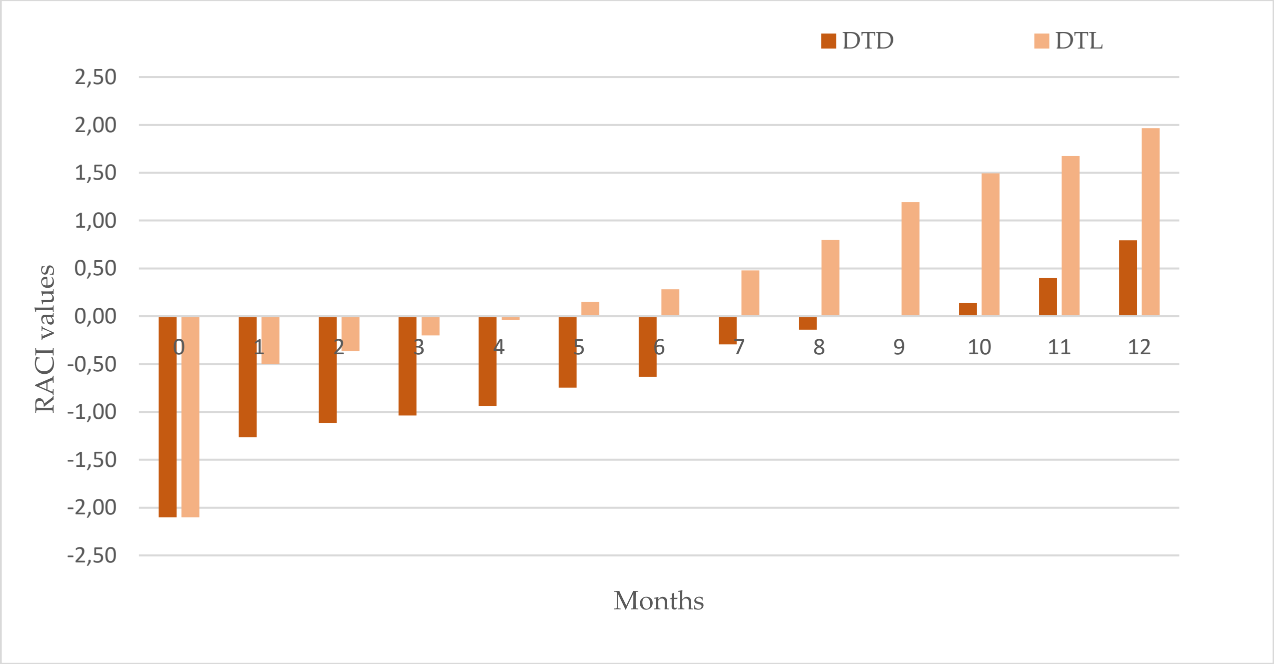

(b)

Figure S1. RACI values of EVOOs (*a*) and dried tomatoes (b) samples.
